# Mycosis Fungoides Involving the Dorsal Tongue

**DOI:** 10.1155/2018/5235246

**Published:** 2018-11-19

**Authors:** Ashley E. Brown, Lindsey Schmidtberger, Kathleeen Kroger, Richard R. Jahan-Tigh, Sarah S. Pinney

**Affiliations:** ^1^McGovern Medical School, University of Texas Health Science Center at Houston, Houston, TX, USA; ^2^Department of Dermatology, University of Texas McGovern Medical School at Houston, Houston, TX, USA

## Abstract

Mycosis fungoides (MF) is a rare cutaneous T-cell lymphoma (CTCL) which can cause significant morbidity. During the disease course, it classically will progress through three clinical stages in the skin: patch-, plaque-, and tumor-stage. The early stages exhibit various histopathological mimics that often lead to misdiagnosis. It rarely affects the oral cavity. Oral MF is historically associated with poor prognosis. We present a rare case of MF afflicting the dorsal tongue and extremities of a 72-year-old male.

## 1. Introduction

Mycosis fungoides, the most common form of CTCL, can cause significant morbidity and mortality. It has many clinical presentations, including patch, plaque, and tumor stage. During the patch and plaque stages, MF closely resembles many common inflammatory dermatoses that dermatologists encounter on a regular basis such as eczema and psoriasis, making early diagnosis difficult. Mycosis fungoides may rarely manifest in the oral cavity in advanced disease. A diagnostic hallmark of MF is epidermotropism, or presence of atypical lymphocytes in the epidermis.

## 2. Case Report

A 72-year-old African American male presented with progressive erythroderma and keratoderma of the palms and soles of unknown etiology for greater than three years. He was originally treated by his primary care physician with topical antifungal cream for a presumed tinea infection without improvement. He presented to the dermatology clinic where multiple biopsies over time were nondiagnostic, revealing nonspecific pathologic diagnoses such as spongiotic dermatitis and psoriasiform dermatitis. He failed to improve after many months of high dose topical steroids and a short course of oral methotrexate.

**Figure 1:**
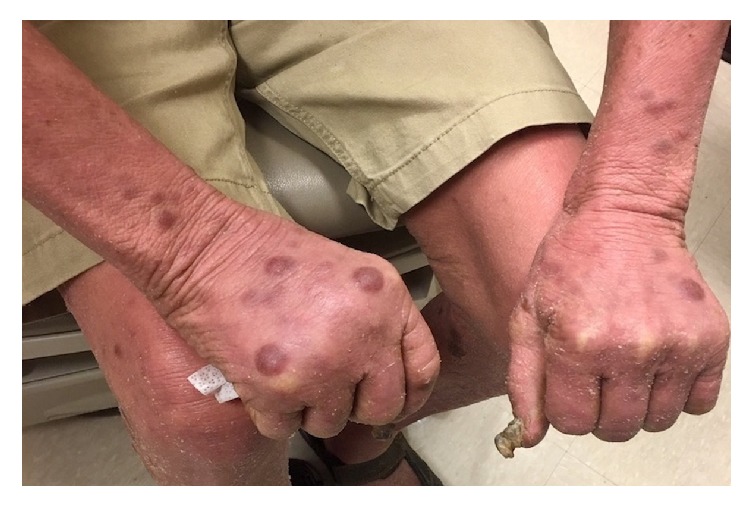
Scattered violaceus nodules, and plaques, some with serous exudate located on the dorsum of the hands.

**Figure 2:**
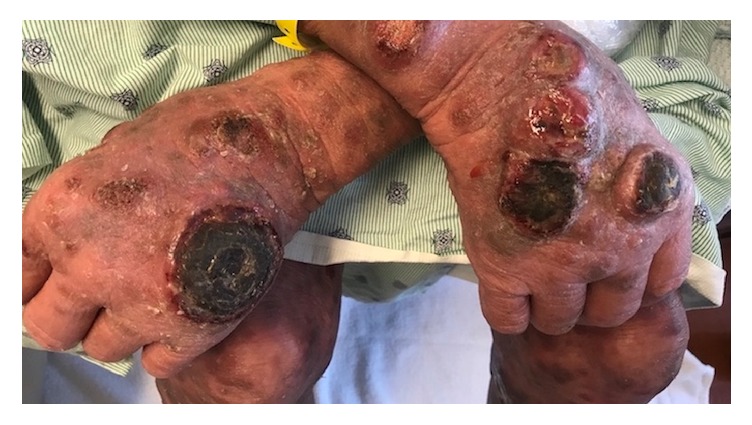
Ulcerated tumors bilaterally on hands.

**Figure 3:**
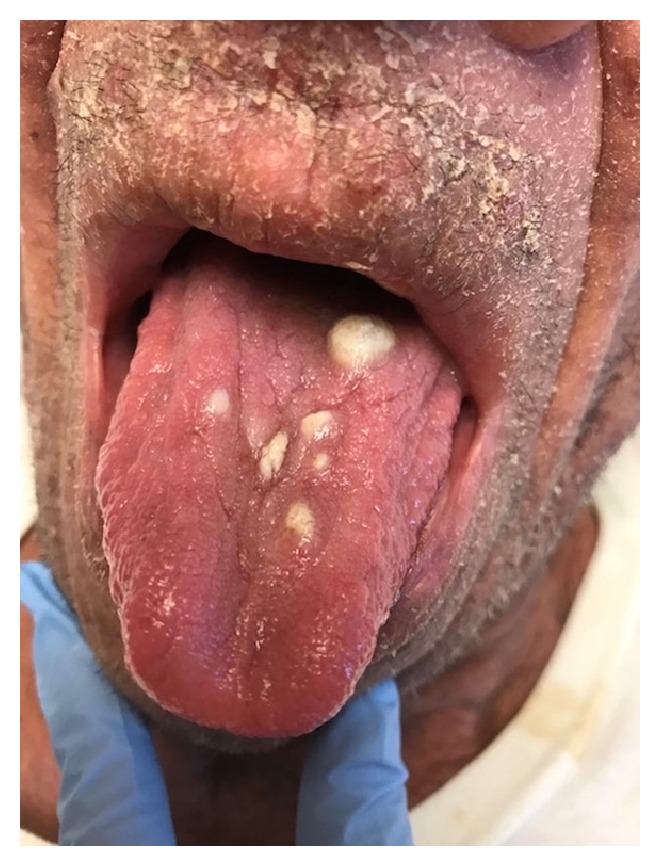
White nodules of the dorsal tongue.

**Figure 4:**
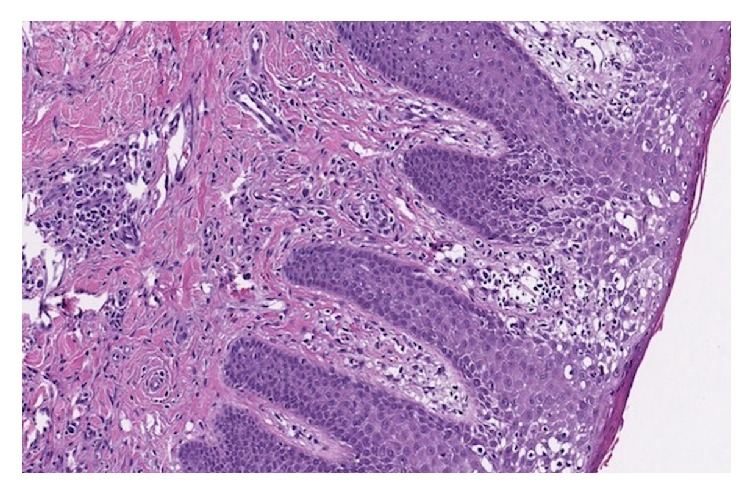
H&E stain of biopsy specimen taken at tumor of right thigh at 10x magnification. A dense dermal atypical lymphoid infiltrate with epidermotropism of atypical lymphocytes into the epidermis.

Approximately one month after methotrexate was discontinued, the patient developed violaceus and erythematous juicy nodules on the cheeks, trunk, and all four extremities (Figure 1). Differential diagnosis included deep fungal infection, acute febrile neutrophilic dermatosis, CTCL, and Kaposi's sarcoma. A biopsy of a large tumor on the right shin was performed and revealed a diffuse infiltrate of atypical inflammatory cells, without epidermotropism, most consistent with MF with large cell transformation. Over the following months, the development of tumors quickly progressed, and many became ulcerated (Figure 2). At this point tumors developed on the dorsal surface of the tongue (Figure 3). At this time, biopsies of the right thigh showed epidermotropism (Figure 4).

## 3. Discussion

The differential diagnosis for erythroderma includes psoriasis, drug reaction, atopic dermatitis (AD), pityriasis rubra pilaris, and CTCL, with AD, drug eruption, and psoriasis being most common. This case highlights the importance of careful monitoring and high level of suspicion with appropriate testing in an erythrodermic patient who is failing to respond to traditional therapies. MF is fairly rare but represents 50% of CTCL cases and can be easily treatable or curable if diagnosed in an early stage [1]. More often MF presents with isolated indolent pink plaques or patches but may present as in our case with erythroderma and subsequent tumor formation. Oral MF is very rare and is associated with advanced disease [2]. Fewer than 60 cases of oral MF have been described. The tongue, palate, and gingiva are affected most frequently among these cases. Cutaneous manifestations typically precede oral MF; however cases have presented with oral findings [2].

The diagnostic histologic finding of epidermotropism found in the patch and plaque stage of MF may also be absent in the tumor stage. Instead histology may show diffuse lymphocytic infiltrate with atypical cells extending as deep as the subcutaneous tissue [3]. Absence of epidermotropism may lead to misdiagnosis with various histopathologically similar conditions such as psoriasis and AD. Various groups have attempted defining an algorithm to better differentiate MF from mimics [4, 5]. Factors that help distinguish MF from benign conditions include long disease duration, pruritus, deterioration of general condition, and epidermotropism [5].

T-cell receptor (TCR) gene rearrangement studies coupled with flow cytometry can be used to support histologic and clinical findings of MF. Identifying a monoclonal TCR gene arrangement by polymerase chain reaction (PCR) of tissue specimens denotes the existence of a dominant clone suggesting malignancy [6]. A study utilizing multiplex PCR/heteroduplex to analyze TCR gene rearrangements of 547 cutaneous biopsies found a 92.7% diagnostic accuracy in CTCL patients [7]. Flow cytometry analysis (FCA) may be performed on peripheral blood samples or adequately prepared biopsy specimens with collagenase. FCA is considered positive for neoplastic T-cell population if there is identification of a homogenous and discrete population of lymphoid cells with a uniform CD4+, CD8+, or CD4-CD8- phenotype and presence of phenotypic abnormality. Abnormalities may consist of absent or reduced CD2, CD5, CD7, or CD26 expression [6].

TCR gene rearrangement studies are particularly important in working up recalcitrant erythroderma in a palmoplantar distribution. Misdiagnosis of palmoplantar MF is common due to clinical similarities to psoriasis, dermatophytic infections, and inflammatory dermatoses. The use of TCR gene rearrangement studies and flow cytometry combined with histopathologic studies can lead to the early diagnosis of MF [8].

## 4. Conclusion

In the absence of classic histopathological findings such as epidermotropism, a diagnosis of MF can take years to establish. A high degree of clinical suspicion should be present when faced with persistent erythroderma that is failing to respond to treatments known to be effective for the condition suspected. Histologic and clinical data can be combined with TCR gene arrangement studies and FCA to support diagnosis. Advanced stage MF may present with oral findings in addition to cutaneous tumors.
